# Haploid biotechnology as a tool for creating a selection material for sugar beets

**DOI:** 10.18699/VJ21.094

**Published:** 2021-12

**Authors:** E.O. Kolesnikova, E.I. Donskikh, R.V. Berdnikov

**Affiliations:** Breeding and Genetic Center “UnionSeedsBeet”, Ltd., VNIISS, Ramonsky district, Voronezh region, Russia; Breeding and Genetic Center “UnionSeedsBeet”, Ltd., VNIISS, Ramonsky district, Voronezh region, Russia; Breeding and Genetic Center “UnionSeedsBeet”, Ltd., VNIISS, Ramonsky district, Voronezh region, Russia

**Keywords:** sugar beet, haploid, doubled haploid, gynogenesis, biotechnology, in vitro, DH lines, сахарная свекла, гаплоид, удвоенный гаплоид, гиногенез, биотехнологии, in vitro, DH-линии

## Abstract

Since the discovery of the phenomenon of haploidy, biotechnology has become an integral part in the successful creation of new varieties and hybrids of various plant species. In particular, these technologies are actively used in agriculture, which is concerned with increasing the volume and improving the quality of products. The integration of haploid production techniques together with other available biotechnological tools such as marker selection (MAS), induced mutagenesis and genetic engineering technologies can significantly accelerate crop breeding. This article shows the main stages in the development of biotechnology since 1921. Now they are successfully used to create doubled haploids to accelerate the selection process of various plants and, in particular, sugar beet, which is the most important sugar crop in regions with a temperate climate. There are several methods for obtaining forms with a single set of chromosomes. For sugar beets, the use of gynogenesis turned out to be expedient, since in this case the other methods turned out to be ineffective in the mass production of haploids. The article considers the stages of obtaining the H and DH lines of Beta vulgaris L., as well as the main stages of biotechnological production of homozygous breeding material of this culture. These stages include selecting parental forms – donor explants, sterilizing buds and introducing non-pollinated ovules in vitro, obtaining haploids, doubling their chromosome set, creating doubled haploids, determining ploidy at different stages, relocating the obtained plants to greenhouses and growing stecklings. A number of advantages that the technology of creating doubled haploids in vitro has in comparison with traditional methods of selection are described. It has been shown that the use of these approaches is relevant when obtaining new highly productive hybrids and varieties of agricultural plants; however, the methods for the production of homozygous forms in sugar beet still require additional research aimed at increasing the efficiency and reproducibility of each stage of the process.

## Abstract (ru)

С момента открытия явления гаплоидии биотехнологии стали неотъемлемой частью в про- цессе успешного создания новых сортов и гибридов различных видов растений. Особенно активно дан- ные технологии применяются в сельском хозяйстве, которое заинтересовано в увеличении объемов и повышении качества производимой продукции. Интеграция приемов получения гаплоидов вместе с дру- гими имеющимися биотехнологическими инструментами, такими как маркерная селекция (MAS), индуци- рованный мутагенез и генно-инженерные технологии, может значительно ускорить селекцию сельскохо- зяйственных культур. В статье показаны основные этапы развития биотехнологий начиная с 1921 г. Теперь они успешно используются при создании удвоенных гаплоидов для ускорения селекционного процесса различных растений, и в частности сахарной свеклы – важнейшей сахароносной культуры в регионах с умеренным климатом. Существует несколько методов получения форм с одинарным набором хромосом. Для сахарной свеклы целесообразным оказалось применение гиногенеза, поскольку остальные приемы были малоэффективны при массовом получении гаплоидов. В публикации рассматриваются этапы полу- чения H- и DH-линий Beta vulgaris L., а также основные этапы биотехнологического производства гомози- готного селекционного материала этой культуры. К ним относятся: отбор родительских форм – доноров эксплантов; стерилизация бутонов и введение неопыленных семяпочек in vitro; получение гаплоидов; удвоение их хромосомного набора; создание удвоенных гаплоидов; определение плоидности на разных этапах; перевод полученных растений в закрытый грунт и выращивание штеклингов. Описан ряд преиму- ществ, которые имеет технология создания удвоенных гаплоидов in vitro по сравнению с традиционными методами селекции. Показано, что применение данных подходов является актуальным при получении но- вых высокопродуктивных гибридов и сортов сельскохозяйственных растений, однако приемы производ- ства гомозиготных форм у сахарной свеклы все еще требуют проведения дополнительных исследований, направленных на увеличение эффективности и воспроизводимости каждого этапа процесса.

## Introduction

Due to the increase in the consumption of agricultural products, an urgent need arose for the development of technologies that accelerate the selection processes of cultivated crops. Sugar beet, which is one of the main industrial crops, is not an exception since sugar consumption is growing every year. Beta vulgaris L. accounts for a significant part of the world’s total sugar production and about 80 % of the obtained beet sugar comes from European countries. At the same time, Russia is the leader in terms of volume in sugar beet cultivation.

The main task of breeding this crop under conditions of intensification of agricultural production is the creation of highly productive domestic hybrids on a linear basis. The process of obtaining promising hybrids is carried out more efficiently when the biotechnological production of new breeding material is introduced. The great economic importance of Beta vulgaris determines considerable interest in optimizing the processes of micropropagation in vitro, creating haploid forms and obtaining homozygous forms on their basis for breeding work. Modern agro-industry should have specialists who are skilled at using biotechnological methods in order to intensify agricultural production and improve its quality.

Homozygous lines are a unique genetic material for accelerating the process of creating new hybrids and reducing its labor intensity, for mapping the populations, for use in functional genomics and molecular selection. All genes of haploid plants are represented by a single allele; due to this fact, all unfavorable recessive signs can be detected at early stages of the selection process. Homozygous lines based on haploids can be obtained within two years whereas classical methods for breeding for heterosis in cross-pollinated crops make it possible to achieve homozygosity only after 6–7 years of inbreeding. Since Beta vulgaris has a 2-year development cycle, in this case the whole process takes on average 12 years. Besides, this culture is characterized by self-compatibility and by the occurrence of inbred depression (Urazaliyev et al., 2013). Nowadays, the production of doubled haploids has become a tool in the selection programs of world research laboratories as an alternative to the classical method of obtaining homozygous lines. Due to the growing understanding of the importance of using such an approach in selection, interest in research in this field is constantly increasing (Datta, 2005). A significant number of new haploid-derived plant varieties are recorded worldwide every year. The EU, Canada, Australia, the USA and China have been leaders in the field of haploid technologies so far (Dunwell, 2010).

## Haploidy

The phenomenon of haploidy has been the object of scientific attention since the beginning of the 20th century and is currently widely known in many angiosperms. The development of experimental haploidy methods began a little later, when the potential of using plants with a single set of chromosomes in creating pure lines for breeding needs was discovered. There are several ways to form haploids:

1. Plants are pollinated by pollen of plants of the same species (inducers of haploids), which are classified as pa-ternal or maternal inducers based on the genetic constitution of the obtained haploids. Respectively, paternal and maternal haploids carry the genome from male and female parents. At the same time, chromosomes-inducers are eliminated in haploid embryonic cells within the first week after pollination (Chaikam et al., 2019).

2. Plants are pollinated by pollen of an unallied species, for example, crossing of wheat with corn. This method is very effective for the production of haploids in most Triticum spp. genotypes, including difficult-to-respond forms in in vitro anther culture. The data obtained by scientists showed that the yield of haploid embryos in individual crosses reached up to 53 % (Djatchouk et al., 2019).

3. Plants are pollinated by pollen of a wild allied species. This method is used in barley selection to obtain haploids when crossing Hordeum vulgare × H. bulbosum (the socalled “bulbosum” method). Elimination of H. bulbosum chromosomes occurs during mitosis and during interphase and is accompanied by the formation of micronuclei and heterochromatinization. Complete elimination of bulb barley chromatin occurs within 5–9 days after pollination. The use of embryo rescue technology (embryo rescue) provides an increase in the efficiency of the method at the next stage and the possibility of its use in selection (Sahijram, Rao, 2015)

4. Pollination by irradiated pollen is a well-documented method of induction of cucumber haploids, which can be obtained from various material such as selection lines, hybrids and varieties. Haploid plants are genetically stable, but it is necessary to double the number of chromosomes in them before further use in selection as it is a very important step. The technology for obtaining doubled haploids of cucumber using pollination with irradiated pollen is considered to be the most developed, convenient, providing a stable yield of DH lines, compared with technologies for obtaining in anthers culture or microspores and non-pollinated ovules in vitro (Gałązka et al., 2015).

5. Several protocols for CENH3-mediated induction of haploids are currently known. However, an effective haploid technology based on CENH3 is not available for crops yet, with the exception of maize, where the haploid level reached 3.6 % (Kelliher et al., 2016). This goal is actively studied now. The expectations for the application of this approach to crops are high, while the creation of viable methods of haploidization based on CENH3 is a difficult task and there is no mass introduction into practice yet (Watts et al., 2016).

6. Obtaining haploids by androgenesis (that means obtaining haploid plants on an artificial nutrient medium from isolated anthers and microspores) and gynogenesis (that means obtaining haploid plants on a nutrient medium from isolated ovules).

During androgenesis, the male gametophyte, under the influence of in vitro inducing factors, switches from the gametophytic path of development to the sporophytic pathway with the formation of an embryoid or androgenic callus, from which regeneration of haploids or doubled haploids is possible. Almost all biotechnological laboratories of breeding and genetic companies in Europe and the USA use anther culture (Touraev et al., 2009; Basu et al., 2010).

When cultivating anthers, callus formation is more common. As a result of further morphogenesis, plants are regenerated from callus cells. The cases of direct embryogenesis, when pro-embryonic structures are formed from immature pollen grains, developing into embryoids that give a rise to haploid plants, are quite rare.

more promising method for obtaining doubled haploids is the culture of isolated microspores, which can be isolated in large quantities, providing potentially embryogenic single haploid cells. This technology is quite simple to implement, cost-effective and provides a high yield of DH plants while optimizing the technology for a specific species and genotype. The absence of somatic tissues in the culture of microspores in vitro makes it possible not to question the origin of the obtained plants (Domblides et al., 2019).

Despite the success achieved in the development of androgenetic methods and the creation of a number of varieties of the most important types of grain crops on their basis, their effective use is hindered by a number of reasons, the main of which is the reproducibility of the results obtained in different seasons for different genotypes in combination with a decrease in the cost of obtaining. A rather high yield of chlorophyll-free seedlings, which affects breeding programs by reducing the frequency of regeneration of green seedlings, is also a problem (Kasha et al., 2001).

In vitro induction of maternal haploids – gynogenesis is used mainly in plants for which androgenesis and pollination induction are ineffective. In contrast to induced

parthenogenesis, in gynogenesis prior to in vitro pollination, ovaries or isolated ovules, rather than defective embryos from seeds, are introduced. When cultured on nutrient media, the haploid cells of the embryo sac form embryoids (direct embryogenesis) or morphogenic callus, from which a plant is formed (indirect embryogenesis). The process of induction of gynogenesis is also influenced by a large number of factors: genotype, growing conditions of the donor plant, stage of gametophyte development, composition of the nutrient medium, and stress. In male-sterile plants, the cultivation of non-pollinated ovules is the only way to obtain haploids. In some plants, such as barley and rice, the induction of green plants is much higher during gynogenesis than in androgenesis. The frequency of the accidental appearance of plants with a single set of chromosomes is insignificant, up to 0.01 %, that is, it is a rare event and has limited practical value (Bohanec, 2009; Kiełkowska et al., 2014).

## History of the development of haploid plant technologies

The first plant identified as a haploid, according to one version, was discovered by A.D. Bergner in 1921. The use of such plants in breeding was suggested by A.F. Blakeslee and J. Belling. The scientists obtained the plants with a single set of chromosomes while trying to mutate Datura stramonium L. by applying cold as a stimulus. The obtained haploid immediately became an innovation among such flowering plants as a sporophyte having a set of chromosomes characteristic of the gametophyte (Blakeslee et al., 1922). Later, the haploid form was identified in the F1 progeny when crossing the species Nicotiana tabacum and N. sylvestris. The obtained plant had some morphological differences from the parental forms, such as long narrow leaves, smaller flowers, sterile pollen, and inability to form mature seeds, which was confirmed by cytological examination (Clausen, Mann, 1924). According to E.F. Gains, a haploid wheat plant that had 21 chromosomes instead of 42 chromosomes as in the parental individuals was discovered in 1925. The haploid form was practically indistinguishable from the diploid form, but it had greater tillering. Distinct differences became noticeable at the time of flowering and during the formation of immature seeds (Gains, Aase, 1926). Later, in the course of research, the induction of haploids in wheat was achieved by pollination of plants with pollen that underwent X-ray irradiation. As a result, male gametes were inactivated and lost the ability to merge with the egg, but stimulated its division and development of the embryo (Katayama, 1934). Studies using X-ray irradiation did not give significant quantitative results and posed a danger to humans.

At the initial stages, haploid forms of plants were obtained by traditional selection methods using distant hybridization. Thus, when Triticum aestivum L. and Secale cereale L. were crossed, two haploids were obtained (Sears, 1988). Later, other methods using various chemicals appeared. With the development of biotechnological techniques, the creation of haploids has become possible for many plant crops.

The haploid form Beta vulgaris L. was first identified by a Swedish scientist A. Levan in the greenhouses of the Swedish Sugar Co Hilleshog breeding station, where in 1942 the scientist treated sugar beet plants with colchicine (Levan, 1945). The collected seeds were germinated and in 1943 the number of chromosomes in the obtained plants was examined. In addition to diploid, triploid and tetraploid plants, one haploid plant was obtained with the number of chromosomes equal to 9, in contrast to the parental forms with 18 chromosomes. A. Levan suggested that colchicine treatment caused damage to gametes: one pollen grain could stimulate the development of the embryo, but was not capable of fertilization. In his study, the author paid special attention to the morphology and cytology of the haploid plant. According to his description, the haploid possessed a large number of narrow leaves, which were smaller in size compared to diploid, triploid and tetraploid plants. According to external signs, the haploid was clearly weaker and lower than diploid plants, but it could form well-developed inflorescences and fertile pollen, which eventually underwent degradation. On the basis of cytogenetic studies, A. Levan concluded that in haploids meiosis was as close as possible to that in diploids, but due to the absence of homologous chromosome pairs the whole mechanism ended in failure. Further research in this field made it possible to obtain experimentally haploid forms of plants with a frequency exceeding the natural level.

In 1964, employees of the Department of Botany of the University of Delhi (India) S. Guha and S.C. Maheswari published data on the results of biochemical studies of meiosis of Datura anthers in culture in vitro (Guha, Maheswari, 1964, 1966). When cultivating mature anthers on nutrient media, scientists discovered embryoids developing from immature microspores. Some of the embryoids that regenerated during the experiment turned into normal seedlings. In further studies, it was found that some of them had a haploid number of chromosomes. Later, the method by which hundreds of haploid plants of various types of tobacco were obtained from pollen grains in vitro was presented. When grown on a nutrient medium, some pollen grains grew into embryonic structures, which, gradually developing, were capable of abundant flowering, but did not form seeds (Nitsch J., Nitsch C., 1969). It also became known that rice haploids (Oryza sativa L.) (Niizeki, Oono, 1968), wheat haploids (Triticum aestivum L.) obtained in anther culture (Ouyang et al., 1973) had been successfully produced in vitro.

In 1982, the culture of isolated microspores became known, which was more effective in the production of haploids (Lichter, 1982). Later, haploids of Triticum aestivum L. were produced in the culture of isolated microspores (Datta, Wenzel, 1987; Tuvesson, Öhlund, 1993), by distant hybridization with wild barley (Hordeum bulbosum L.) and corn (Zea mays L.) (Barclay, 1975; Laurie, Bennett, 1986; Inagaki, Tahir, 1990).

The extraction of anthers from buds and their subsequent opening to release microspores was a rather laborious procedure that was improved by M. Zheng in the course of obtaining haploids and doubled haploids from wheat microspores. The developed technology included the stages of homogenization, filtration, and centrifugation of the obtained sample in a density gradient. As a result, M. Zheng was able to collect a fraction of viable embryogenic microspores for culturing on nutrient media under in vitro conditions, which allowed to optimize the method of wheat microspore extraction (Zheng, 2003). Further, the work of L. Cistué and Z. Labbani with co-authors on the DH protocol for durum wheat was published (Cistué et al., 2006; Labbani et al., 2007). The main improvements that they applied in their work were pretreatment with mannitol and the use of colchicine in vitro.

So, with the acquisition of more and more data on the possibility of creating haploid forms of higher plants in vitro, the value of their use in breeding and the importance of the development of biotechnological methods were revealed. By now, for many plant crops, such as wheat, triticale, barley, rice, corn, cabbage, carrots, etc., effective techniques have been developed that make it possible to obtain haploid plants to create clean lines.

## Development of sugar beet haploid biotechnologies

In 1971, N. Bosemark reported the production of five haploids by pollinating plants with wild beet pollen and irradiated sugar beet pollen. In addition to the emergence of haploid forms, it was possible to create homozygous diploid and tetraploid lines after treating previously germinated seeds with colchicine (Bosemark, 1971). In 1983, it became known that haploids could be obtained by the method of distant hybridization. When sterile sugar beet plants were pollinated with red table beet pollen, the incidence of haploids was 0.013 % (Seman, 1983). These methods were aimed at stimulating an unfertilized egg to develop, however, they showed insignificant results in the number of haploids obtained, which was insufficient for large-scale breeding work.

The androgenesis pathway for producing sugar beet haploids has generally been found to be ineffective. Anthers most often induced callus, proembryogenic structures, and roots on the mineral media used; however, their percentage depended on the combination of growth substances used. According to the results of the study by J. Rogozinska and M. Goska, the Linsmeier and Skoog medium with the addition of zeatin 6-(4-hydroxy-3-methyl-trans-2-butenylamino) purine) or zeatin and NAA (1-naphthaleneacetic acid) was recognized as the best medium for differentiation acid, and the addition of PFP (p-fluorophenylalanine) increased the percentage of anther differentiation (Rogozinska, Goska, 1982). In addition to callus and roots, vegetative buds were formed on one anther out of approximately 140,000 tested, from which numerous diploid plants were obtained. Cytological analyzes showed the formation of multicellular structures, which subsequently degenerated. In other similar experiments, whole plants developed, but their tissue was more often diploid, and the gametophytic origin of the regenerants was not confirmed (Gürel E. et al., 2008). In recent studies, conditions have been developed for the direct induced androgenesis of sugar beet in in vitro culture. The process of obtaining haploids included cold pretreatment of explants, which was a necessary factor in initiating the transition of microspores from the gametophytic to the sporophytic pathway. For the cultivation of anthers and developed embryoids, the composition of the medium was modified, which included 2,4-D and 6-BAP. As a result of the experiment, from 0.15 to 1.32 % of microclones of androgenic origin were obtained (Hontarenko, Herasymenko, 2018). However, it was not effective enough for mass production of haploid forms. A lot of studies in this field gave unsatisfactory results. The morphogenetic response of in vitro cultivation of elements of the male generative system of B. vulgaris, according to data available today, is considered very low.

D. Hosemans and D. Bossoutrot were the first to succeed in obtaining sugar beet haploids by cultivating nonpollinated ovules. In their experiment, they identified 0.17 % of haploids formed (Hosemans, Bossoutrot, 1983; Bossoutrot, Hosemans, 1985). Their further histological study showed that the regenerated embryoids could have originated from an unfertilized egg or antipode. However, the resulting gynogenetic plants showed the phenomenon of endopolyploidy at the level of the root meristem, while the shoot meristem remained haploid. From the moment when it became clear that this approach may be the only effective method for producing haploid sugar beet plants, numerous in vitro studies have begun to optimize this process.

Interest in sugar beet gynogenesis increased every year, and in 1987 J. Van Geyt and his co-authors reported the production of haploids from ovules with a frequency of up to 6.1 % (Van Geyt et al., 1987). The results of histological studies in the experiment confirmed that the obtained plants originated from haploid cells of the embryo sac, but spontaneous polyploidization was observed at the root tips, as in the study of D. Hosemans and D. Bossoutrot (Hosemans, Bossoutrot, 1983; Bossoutrot, Hosemans, 1985). According to J. Van Geyt, the shape of the extracted ovules was of great importance when introduced into tissue culture. It was found that the loss of the shape of the comma by the explant was accompanied by the death of the egg. It was also reported that plant regeneration was inhibited by the formation of callus from the mother’s tissue, but after removing it and transferring the ovule to a new nutrient medium containing charcoal, its reappearance could be suppressed. Further studies of gynogenesis revealed the dependence of this process on various conditions.

In the course of M. Doctrinal’s studies, it was found that factors such as the nature and concentration of hormones used, cultivation temperature, seasonal effects, and genotype had a great influence on the development of haploid plants (Doctrinal et al., 1989). According to the results of observations, for the initiation of embryoidogenesis in nonpollinated ovules of sugar beet, the most preferable temperature was 27 °C, while the seasonal effect had a significant impact. The most active regeneration was observed in July. It was also found that the hormonal composition of nutrient media influenced the pathways of female gametophyte morphogenesis and the formation of morphological structures. The best qualitative and quantitative indicators of the gynogenic response in the studied genotypes were observed on nutrient media containing 2.85 μM 3-indoleacetic acid and 0.88 μM 6-benzylaminopurine, as well as on a medium containing 2.3 μM kinetin. Depending on the genotype, when using these media, from 6 to 10 % of viable plants were obtained, 81 % of which turned out to be haploids. Research by M. Doctrinal confirmed the promise of obtaining sugar beet haploids by gynogenesis. The development of biotechnological methods continued, taking into account many factors affecting the gynogenesis of Beta vulgaris L. in vitro, aimed at optimizing the cultivation conditions and increasing the efficiency of the corresponding methods.

In the works of H. Lux and co-authors, it was noted that the yield of regenerants from the ovules decreased from the most active regeneration in September to the lowest in January (Lux et al., 1990). These data suggested that the effectiveness of gynogenesis was seasonal. According to the authors, despite the laboriousness of the process, gynogenesis turned out to be a more suitable method for obtaining sugar beet haploids in vitro, as the species was not amenable to androgenesis. Depending on the genotype, from 0 to 13 % of haploid plants were obtained, while in 10 % of plants during cultivation and reproduction, spontaneous doubling of chro-mosomes was observed while 90 % remained haploids.

For the successful application of gynogenesis in practical problems, the number of formed haploid plants is of great importance. S. Gürel and co-authors confirmed that cold pretreatment and the action of activated charcoal can increase the incidence of embryo formation (Gürel S. et al., 2000). In the experiments of scientists, embryos that developed from an egg formed shoots with a haploid number of chromosomes. However, when developing optimal conditions for obtaining haploids, the problem of genotypic dependence of the response to cultivation conditions arose. The yield of the obtained embryoids differed in sugar beet lines, as well as the response of developed microclones to different growth conditions. Genotypic differences in the response to cultivation conditions are a serious problem not only in the culture of ovules (Hansen et al., 1995), but also in the work with other tissues of sugar beet (Mikami et al., 1989; Gürel E., 1997); therefore, it is recommended to select the composition of the nutrient medium, as well as the cultivation conditions, for each genotype individually.

Studies conducted by many scientists have indicated the importance of pre-cold treatment of the material for increasing the rate of gynogenesis of Beta vulgaris (Lux et al., 1990; Gürel S. et al., 2000; Svirshchevskaya, Dolezel, 2000; Pazuki et al., 2018a). H. Lux and co-authors showed that cold treatment (4 °C) for 4–5 days was able to significantly increase the rate of sugar beet gynogenesis. An increase in the period of cold exposure to inflorescences over one week reduced the gynogenic response of ovules, while preliminary cold treatment for 7 days and the use of BAP at a concentration of 1 mg/L had a stimulating effect when switching the development of explants from the gametophytic to the sporophytic pathway. It was emphasized that the influence of the season and genotype on the rate of regeneration was significant. In addition to cold pretreatment, the percentage of regeneration also increased when the spectrum of light irradiation, used in the cultivation of ovules in a thermal room, was shifted towards the red region of the spectrum (D’Halluin, Keimer, 1986).

Researchers E. Weich and M. Levall (2003) proposed a protocol for obtaining doubled sugar beet haploids, in which all stages of creating breeding material were considered: growing donor plants, collecting inflorescences, surface sterilization of the material, isolating ovules, cultivating haploid embryoids, reproduction of haploids, obtaining doubled haploids, their rooting, transfer to greenhouse conditions, and acclimatization. The conditions for each stage were described, recommendations were given for manipulating plant material, the compositions of the optimal nutrient media were described. This protocol was of a recommendatory nature; due to the diversity of the genotypic response to cultivation conditions, it should be modified for different genotypes of sugar beet.

To obtain haploids efficiently, M. Tomaszewska-Sowa described a two-stage cultivation process, in which the explants were non-pollinated ovules isolated after sterilization from the buds of the generative shoots of sugar beet plants. The reproductive structures were kept in a liquid nutrient medium for 12 weeks (Tomaszewska-Sowa et al., 2017). The regenerated explants were transferred to a solid nutrient medium with a modified composition, after which the formation of shoots was observed. It was found that the organogenesis of the ovules in the two-phase method was not direct, but proceeded through the formation of callus tissue. The efficiency of regeneration depended on the type and origin of the explant. Differentiation processes in somatic embryoids were enhanced by the presence of 6-BAP and 2,4-D in the medium, which, in turn, increased the number of formed specialized tissue structures.

The ratio of hormones in the substrate has a major influence on the pathways of development of the embryonic structure. At present, the protocols for obtaining sugar beet haploids require further development and improvement, therefore, studies on the selection of the optimal composition of media and the search for other factors stimulating gynogenesis are still ongoing.

## Stages of creating homozygous material in vitro

Selection of parental forms

So, the selection of donor plants can be considered the first stage with which the work on the introduction of plant material in vitro begins. To reduce the level of infection of explants, the collection of inflorescences should be carried out in dry weather, with a prolonged absence of precipitation. This procedure is best done at the beginning of the flowering period. Depending on the region of growth, it can be May–July. The collected inflorescences of each genotype are placed in plastic bags, labeled and stored in the cold until the stage of sterilization of plant material under laboratory conditions.

Sterilization of material with the introduction of ovules in vitro

Plants are easily affected by various epiphytic and endophytic microorganisms and viruses. That is why the stage that determines the success of the in vitro material introduction process is the high-quality sterilization of the starting material. Previously developed techniques for sterilizing explants using a number of mercury-containing preparations (mercury chloride, diocide, merthiolate) were found to be effective (Granda, 2009), but very toxic to both humans and plants. Over time, they were supplanted by techniques using other substances. For surface sterilization, plant tissues can be treated with chlorine-containing substances (calcium or sodium hypochlorite, bleach, chloramine), hydrogen peroxide, ethanol. O. Jones suggested using solutions containing sodium hypochlorite (Domestos) as a disinfectant for sterilization (Jones et al., 1979). E. Weich and M. Levall used the commercial product Klorin or 3 % sodium hypochlorite for sterilization. After sterilization, the material was thoroughly washed with distilled water and stored in a refrigerator at 8 ± 2 °C (Weich, Levall, 2003). When choosing a sterilization method, it is necessary to take into account both its effectiveness against bacterial and fungal infections, and the prevention of damage to plant tissues. The use of new sterilizing agents increases the likelihood of an effective explant sterilization process.

Introduction of ovules into culture in vitro

By now, despite the emergence of high-tech devices and devices that facilitate some manipulations in laboratories, the creation of haploid material depends on the delicate manual work of the operator. The process of extraction of sugar beet ovules when introduced into in vitro culture has been described in detail by A. Pazuki et al. (Pazuki et al., 2018b). The scientists performed this process under sterile conditions under a stereomicroscope using tweezers and a scalpel. The first closed and subsequent buds were opened towards the top of the inflorescence, introduced into Petri dishes containing the nutrient medium. The cultivation was carried out at a temperature of 27 ± 2 °C with an 18-hour photoperiod. Regenerated ovules were transferred to MS proliferation and propagation medium containing 0.2 mg/L of kinetin, 10 g/L of sucrose. Due to the different reaction of genotypes, the composition of the nutrient media used and the cultivation conditions may be different for each genotype at all stages of creating the breeding material. The regeneration of the ovules can begin as early as 2–3 weeks after injection. The developed full-fledged microclones are further maintained in in vitro culture

Determination of ploidy of the obtained regenerant

Phenotypically, haploid plants differ from diploid plants in height, size and number of organs and have narrower leaf plates; however, visual determination of ploidy does not give accurate results. After the formation of normally developed regenerants from non-pollinated ovules, their ploidy is checked, since there is a possibility of obtaining seedlings from their somatic cells that have a diploid or mixoploid chromosome set.

One of the reliable methods for determining ploidy is the counting of chromosomes which are at the stage of mitosis of actively growing tissues (growth zones of the root or young leaves). The method is laborious and requires lengthy preparation. The process of counting chromosomes in sugar beet cells is described in detail by A. Pazuki and coauthors (Pazuki et al., 2018a). Young leaves of seedlings were treated in vitro for 3 hours with a solution of 8-hydroxyquinoline (0.002 M), followed by their fixation in a freshly prepared solution of 96 % ethanol and hydrochloric acid (2:1). Then they were washed and stored in distilled water. Then a small piece of leaf tissue was transferred into a drop of 3 % orsein in 45 % acetic acid onto a glass slide and crushed under a cover glass. Then the chromosomes were counted under a light microscope.

An alternative to the laborious method of counting chromosomes under a microscope has become flow cytometry of cell nuclei, which is a more convenient and a faster way to determine ploidy. This hardware method is based on measuring the amount of DNA in the nuclei of cells in the mode of one-by-one analysis in a liquid flow using signals of light scattering and fluorescence, and has high accuracy and productivity (Galbraith, 2010). The flow cytometry method allows to determine the ploidy of microclones in a short time without causing significant damage to the studied plants, which is important when working with a limited volume of regenerated material.

Doubling the number of chromosomes

The main goal of induction of gynogenesis is to obtain haploids to create clean lines. For this, at the next stage, the number of chromosomes in normally developed haploids should be doubled in vitro or in vivo by methods. A. Hansen and co-authors studied the effectiveness of antimitotic agents directly in the culture of sugar beet ovules (Hansen et al., 1998). According to the results of the experiment, amiprofosmethyl showed relatively low toxicity to the embryo and contributed to obtaining an average of 4.7 diploid plants per 100 injected explants. According to S. Gürel, the most effective method for creating doubled haploids is the treatment of plants with antimitotic agents such as colchicine, oryzalin, trifluralin or short-term cultivation of shoots on nutrient media containing these substances. Colchicine is the most commonly used alkaloid, which is able to inhibit the formation of the fission spindle at the prophase stage and to stop the separation of chromosomes to the poles of daughter cells. Violation of this process leads to a doubling of the number of chromosomes in the mother’s cell. S. Gürel and co-authors (2000) showed in their studies that treatment of haploids with colchicine and trifluralin gave similar results, and both agents were more effective when used in the form of liquid solutions than when added to an agar medium. For successful diploidization, scientists immersed the grown haploids in a liquid MS medium containing 150 mg/L of an antimitotic agent (colchicine), 1 mg/L of BAP, and 3 % sucrose for a period of 48 hours at a temperature of 27 °C. After treatment, the shoots were washed with sterile distilled water and transferred onto solid MS medium supplemented with 1 mg/L BAP. The number of plants with a doubled chromosome set reached 29.1 %.

To obtain DH plants, E. Weich and M. Levall first removed the tips from the roots of haploid plants, and then incubated them for 5 hours in a solution of 0.2 % colchicine and 0.25 % DMSO (Weich, Levall, 2003). A similar technique was also used with a 0.3 % colchicine solution, in which the regenerants were immersed in the root system for 24 hours, and then they were planted in the soil mixture. At the same time, 19 % of the treated haploid plants doubled the set of chromosomes (Svirshchevskaya, Dolezel, 2000). It has also been reported that applying a 0.1 % colchicine solution with 2 % DMSO to the meristem of a sugar beet haploid once a day for 3 days would result in the doubling of the chromosome set (D’Halluin, Keimer, 1986). M. Ragot and P. Steen (1992) placed a cotton swab moistened with 0.2 % colchicine solution on the apical buds of potted haploid plants for three days and got up to 50 % doubled haploids.

It should be noted that after the treatment of haploids with antimitotic agents, some time later, repeated analysis of the ploidy of microclones and careful selection of the obtained doubled haploids from the entire volume of experimental material will be necessary.

In modern breeding programs, molecular genetic methods are additionally used to determine the valuable agricultural properties of the obtained forms. These methods are usually aimed at identifying stress resistance genes and target alleles responsible for encoding a certain trait. This makes it possible to significantly reduce the breeding process and accumulate the desired alleles in one genotype. Marker assisted selection (MAS) has become the main molecular selection method, which is widely used in the breeding of many crops (Muranty et al., 2014). Genetic markers can be used at various stages of the biotechnological process and breeding programs in order to select promising genotypes.

Rooting of doubled haploids and obtaining stecklings

The next stage is the rooting of the obtained plants of doubled haploids in vitro in order to increase their survival when adapting to outdoor conditions. During the period of preparation of doubled haploids for planting in greenhouse conditions, one may also encounter a low frequency of root formation. This process under in vitro conditions can be delayed by bright lighting, required by the green part of the microclones, by a high concentration of salts and carbohydrates or by the presence of hormones and a low concentration of oxygen in the nutrient medium. Due to the different genotypic response, the search for optimal nutrient substrate composition and microclone culture conditions continues.

Normally developed DH microclones with a developed root system are planted in the greenhouses. Due to the transplantation from in vitro conditions, the stage of adaptation of the planted plants lasts up to 4 weeks; meanwhile, a gradual decrease in air humidity is carried out in the greenhouse. Subsequently, after 2–3 months of growing, the stecklings are formed and the plants will be ready for harvesting. The biotechnological cycle of creating a new homozygous material ends with the stage of artificial vernalization of the stecklings at low temperatures. After that, the homozygous material is sent to further stages of the breeding process, it is planted in experimental field conditions in order to grow flowering plants and carry out crosses

## Conclusion

Thanks to the introduction of biotechnology, the process of creating new sugar beet hybrids can be significantly accelerated. Obtaining doubled haploids can significantly reduce the time and resources spent on creating clean lines. The most successful method for obtaining Beta vulgaris L. haploids is the method of induced gynogenesis that implies the cultivation of non-pollinated ovules in vitro with the subsequent formation of plants with a haploid set of chromosomes. To create DH lines, doubling of the number of haploid chromosomes using antimitotic agents, ploidy control of the created material, and growing of microclones in greenhouse conditions are used. The biotechnological stage ends with the production of doubled haploid plant plugs.

Processes in tissue culture of sugar beet, in particular the induction of H and DH forms, still require additional research. Analysis of the scientific literature shows that in order to maximize efficiency and reproducibility, the production of doubled haploids Beta vulgaris L. needs to be studied more thoroughly and be improved using new approaches. Biotechnology laboratories need to be able to obtain haploids in a sufficiently large amount. Therefore, the methods of cultivating explants, diploidization, rooting, and adaptation of regenerants when transplanted from sterile in vitro conditions into soil, require an increase in efficiency. Whereas improving each stage of the process is still an urgent task, the ultimate goal will be to increase the quality and volume of the output of the finished homozygous material.

## Conflict of interest

The authors declare no conflict of interest.

## References

Barclay I.R. High frequencies of haploid production in wheat (Triticum
aestivum) by chromosome elimination. Nature. 1975;256:
410-411.

Basu S.K., Eudes F., Kovalchuk I. Role of recA/RAD51 gene family
in homologous recombination repair and genetic engineering of
transgenic plants. In: Kumar A., Sopory S. (Eds.) Applications of
Plant Biotechnology: In vitro propagation, plant transformation
and secondary metabolite production. Ch. 12. India, New Delhi:
I K Int. Publ. House Pvt. Ltd., 2010;231-255.

Blakeslee A.F., Belling J., Farnham M.E., Bergner A.D. A haploid
mutant in the Jimson Weed, “Datura stramonium”. Science. 1922;
55(1433):646-647. DOI 10.1126/science.55.1433.646.

Bohanec B. Doubled haploids via gynogenesis. In: Touraev A.,
Foster B.P., Jain E.M. (Eds.) Advances in Haploid Production in
Higher Plants. Dordrecht: Springer Science + Business Media BV,
2009;35-46. DOI 10.1007/978-1-4020-8854-4.

Bosemark N.O. Haploids and homozygous diploids, triploids and
tetraploids in sugar beet. Hereditas. 1971;69(2):193-204. DOI
10.1111/j.1601-5223.1971.tb02433.x.

Bossoutrot D., Hosemans D. Gynogenesis in Beta vulgaris L.:
from in vitro culture of unpollinated ovules to the production of
doubled
haploid plants in soil. Plant Cell Rep. 1985;4:300-303.
DOI 10.1007/BF00269883

Chaikam V., Molenaar W., Melchinger A.E., Boddupalli P.M.
Doubled
haploid technology for line development in maize:
technical advances and prospects. Theor. Appl. Genet. 2019;132:
3227-3243. DOI 10.1007/s00122-019-03433-x.

Cistué L., Soriano M., Castillo A.M., Vallés M.P., Sanz J.M., Echavarri
B. Production of doubled haploid in durum wheat (Triticum
turgidum L.) through isolated microspore culture. Plant Cell Rep.
2006;25(4):257-264. DOI 10.1007/s00299-005-0047-8.

Clausen R.E., Mann M.C. Inheritance in Nicotiana tabacum: V. The
occurrence of haploid plants in interspecific progenies. Proc.
Natl. Acad. Sci. USA. 1924;10(4):121-124. DOI 10.1073/pnas.
10.4.121.

Datta S.K. Androgenic haploids: factors controlling development
and its application in crop improvement. Curr. Sci. 2005;89(11):
1870-1878. DOI 10.1007/978-94-017-1203-319.

Datta S.K., Wenzel G. Isolated microspore derived plant formation
via embryogenesis in Triticum aestivum L. Plant Sci. 1987;48:
49-54.

D’Halluin K., Keimer B. Production of haploid sugarbeets (Beta vulgaris
L.) by ovule culture. In: Horn W., Jensen C.J., Odenbach W.,
Schieder O. (Eds.) Genetic Manipulation in Plant Breeding. Berlin:
Walter de Gruyter & Co., 1986;307-309.

Djatchouk T.I., Akinina V.N., Khomyakova O.V., Kalashnikova E.V.
Distant hybridization as a method of haploid production in cereals.
Biotekhnologiya i Seletsiya Rasteniy = Plant Biotechnology
and Breeding. 2019;2(2):44-52. DOI 10.30901/2658-6266-2019-2-
44-52. (in Russian)

Doctrinal M., Sangwan R.S., Sangwan-Norreel B.S. In vitro gynogenesis
in Beta vulgaris L.: effects of plant growth regulators, temperature,
genotypes and season. Plant Cell Tissue Organ Cult.
1989;17:1-12. DOI 10.1007/BF00042276.

Domblides E.A., Belov S.N., Soldatenko A.V., Pivovarov V.F. Production
of doubled haploids in cucumber. Ovoshchi Rossii = Vegetable
Crops of Russia. 2019;5:3-14. DOI 10.18619/2072-9146-
2019-5-3-14. (in Russian)

Dunwell J.M. Haploids in flowering plants: origins and exploration.
Plant Biotechnol. J. 2010;8(4):377-424. DOI 10.1111/j.1467-
7652.2009.00498.x.

Gains E.F., Aase H.C. A haploid wheat plant. Am. J. Bot. 1926;13(6):
373-385.

Gałązka J., Słomnicka R., Góral-Radziszewska K., Niemirowicz-Szczytt
K. From pollination to DH lines – verification and optimization
of protocol for production of doubled haploids in cucumber.
Acta Sci. Pol. Hortorum Cultus. 2015;14(3):81-92.

Galbraith D.W. Flow cytometry and fluorescence-activated cell
sorting in plants: the past, present, and future. Biomédica. 2010;
30(Suppl.):
65-70. DOI 10.7705/biomedica.v30i0.824

Granda H.R.K. Micropropagation of chrysanthemums. Izvestiya Timiryazevskoy
Selskokhozyaystvennoy Akademii = Bulletin of the
Timiryazev
Agricultural Academy. 2009;1:145-148. (in Russian)

Guha S., Maheshwari S. In vitro production of embryos from anthers
of Datura. Nature. 1964;204:497. DOI 10.1038/204497a0.

Guha S., Maheshwari S. Cell division and differentiation of embryos
in the pollen grains of Datura in vitro. Nature. 1966;212:97-98.
DOI 10.1038/212097a0.

Gürel E. Callus and root development from leaf explants of sugar
beet (Beta vulgaris L.): variability at variety, plant and organ level.
Turk. J. Botany. 1997;21:131-136.

Gürel E., Gürel S., Lemaux P.G. Biotechnology applications for sugar
beet. Crit. Rev. Plant Sci. 2008;27(2):108-140. DOI 10.1080/
07352680802202000.

Gürel S., Gürel E., Kaya Z. Doubled haploid plant production from
unpollinated ovules of sugar beet (Beta vulgaris L.). Plant Cell
Rep. 2000;19(12):1155-1159. DOI 10.1007/s002990000248.

Hansen A.L., Gertz A., Joersbo M., Andersen S.B. Short-duration
colchicine treatment for in vitro chromosome doubling during
ovule culture of Beta vulgaris L. Plant Breed. 1995;114(6):515-
519. DOI 10.1111/j.1439-0523.1995.tb00847.x.

Hansen A.L., Gertz A., Joersbo M., Andersen S.B. Antimicrotubule
herbicides for in vitro chromosome doubling in Beta vulgaris L.
ovule culture. Euphytica. 1998;101(2):231-237. DOI 10.1023/
a:1018380103304.

Hontarenko S.M., Herasymenko H.M. Direct induced androgenesis
in culture in vitro in sugar beet (Beta vulgaris). Plant Varieties
Studying and Protection. 2018;14(4):375-381. DOI 10.21498/
2518-1017. 14.4.2018.151900. (in Ukranian)

Hosemans D., Bossoutrot D. Induction of haploid plants from in vitro
culture of unpollinated beet (Beta vulgaris L.). Z. Pflanzenzücht.
1983;91(1):74-77.

Inagaki M.N., Tahir M. Comparison of haploid production frequencies
in wheat varieties crossed with Hordeum bulbosum L.
and maize. Jpn. J. Breed. 1990;40(2):209-216. DOI 10.1270/
jsbbs1951.40.209.

Jones O.P., Pontikis C.A., Hopgood M.E. Propagation in vitro of five
apple scion cultivars. J. Hortic. Sci. 1979;54(2):155-158. DOI
10.1080/00221589.1979.11514863.

Kasha K.J., Simion E., Oro R., Yao Q.A., Hu T.C., Carlson A.R. An
improved in vitro technique for isolated microspore culture of
barley. Euphytica. 2001;120:379-385. DOI 10.1023/A:1017564
100823.

Katayama Y. Haploid formation by X-rays in Triticum monococcum.
Cytologia. 1934;5(2):235-237.

Kelliher T., Starr D., Wang W., McCuiston J., Zhong H., Nuccio
M.L., Martin B. Maternal haploids are preferentially induced
by CENH3-tailswap transgenic complementation in maize. Front.
Plant Sci. 2016;7:414. DOI 10.3389/fpls.2016.00414.

Kiełkowska A., Adamus A. Baranski R. An improved protocol for
carrot haploid and doubled haploid plant production using induced
parthenogenesis and ovule excision in vitro. In Vitro Cell.
Dev. Biol. Plant. 2014;50(3):376-383. DOI 10.1007/s11627-014-
9597-1.

Labbani Z., Buyser J.D., Picard E. Effect of mannitol pretreatment
to improve green regeneration on isolated microspore culture in
Triticum turgidum ssp. durum cv. ‘Jennah Khetifa’. Plant Breed.
2007;126(6):565-568. DOI 10.1111/j.1439-0523.2007.01399.x.

Laurie D.A., Bennett M.D. Wheat × maize hybridization. Can. J.
Genet. Cytol. 1986;28(2):313-316. DOI 10.1139/g86-046.

Levan A. A haploid sugar beet after colchicine treatment. Hereditas.
1945;31:399-410. DOI 10.1111/j.1601-5223.1945.tb02760.x.

Lichter R. Induction of haploid plants from isolated pollen of Brassica
napus. Z. Pf lanzenphysiol. 1982;105(5):427-434. DOI
10.1016/S0044-328X(82)80040-8.

Lux H., Herrmann L., Wetzel C. Production of haploid sugar beet
(Beta vulgaris L.) by culturing unpollinated ovules. Plant Breed.
1990;104:177-183. DOI 10.1111/j.1439-0523.1990.tb00420.x.

Mikami T., Sudoh R. Nagao E., Kinoshita T. Genotypic variation in
the in vitro morphogenesis from leaf explants of Beta vulgaris L.
and Beta maritima L. Euphytica. 1989;40:271-273. DOI 10.1007/
BF00024523.

Muranty H., Jorje V., Bastien C., Lepoittevin C., Bouffier L., Sanchez
L. Potential for marker-assisted selection for forest tree
breeding: lessons from 20 years of MAS in crops. Tree Genet.
Genomes. 2014;10:1491-1510. DOI 10.1007/s11295-014-0790-5.

Niizeki H., Oono K. Induction of haploid rice plant from anther culture.
Proc. Jpn. Acad. 1968;44(6):554-557. DOI 10.2183/pjab
1945.44.554.

Nitsch J.P., Nitsch C. Haploid plants from pollen grains. Science.
1969;163(3862):85-87. DOI 10.1126/science.163.3862.85.

Ouyang Y.W., Hu C.C., Chuang C.C., Tseng C.C. Induction of pollen
plants from anthers of Triticum aestivum L. cultured in vitro. Sci.
Sin. 1973;16:79-95.

Pazuki A., Aflaki F., Gürel E., Ergül A., Gürel S. Gynogenesis induction
in sugar beet (Beta vulgaris) improved by 6-benzylaminopurine
(BAP) and synergized with cold pretreatment. Sugar Tech.
2018a;20:69-77. DOI 10.1007/s12355-017-0522-x.

Pazuki A., Aflaki F., Gürel S., Ergül A., Gürel E. Production of
doubled haploids in sugar beet (Beta vulgaris): an efficient method
by a multivariate experiment. Plant Cell Tissue Organ Cult.
2018b;132(1):85-97. DOI 10.1007/s11240-017-1313-5.

Ragot M., Steen P. Genetic and environmental effects on chromosome
doubling of sugarbeet (Beta vulgaris L.) haploids. Euphytica.
1992;63:233-237. DOI 10.1007/BF00024549.

Rogozinska J.H., Goska M. Attempts to induce haploids in anther
culture of sugar, fodder and wild species of beet. Acta Soc. Bot.
Pol. 1982;51(1):91-105.

Sahijram S., Rao B.M. (Eds.). Hybrid Embryo Rescue in Crop Improvement.
Plant Biology and Biotechnology. Division of Biotechnology,
Indian Institute of Horticultural Research (IIHR).
India: Bangalore, 2015;2:363-383. DOI 10.1007/978-810322-
2283-5_18.

Sears E.R. History of Chinese Spring aneuploids. In: Proc. 7th Int.
Wheat Genetics Symp. 1988;1:4-6.

Seman I. Possibilities of detection and induction of haploids in Beta
vulgaris L. Biologia (Bratislava). 1983;38(11):1113-1122.

Svirshchevskaya A.M., Dolezel FJ. Production and performance of
gynogenetic
sugarbeet lines. J. Sugar Beet Res. 2000;37(4):117-
133. DOI 10.5274/jsbr.37.4.117.

Tomaszewska-Sowa M., Figas A.S., Gatz A. Histological analysis of
organogenesis and somatic embryogenesis during shoot formation
in sugar beet (Beta vulgaris L.) via gynogenesis. Polish J. Nat.
Sci. 2017;32(4):705-717.

Touraev A., Forster B.P., Jain S.M. (Eds.) Advances in Haploid Production
in Higher Plants. Dordrecht: Springer Science + Business
Media BV, 2009;347. DOI 10.1007/978-1-4020-8854-4.

Tuvesson I.K.D., Öhlund R.C.V. Plant regeneration through culture
of isolated microspores of Triticum aestivum L. Plant Cell Tissue
Organ Cult. 1993;34:163-167.

Urazaliyev K.R., Orsini J.M., Abekova A.M., Bazylova T.A.,
Daniyarova
A.K. Speeding wheat breeding using dihaploids obtained
by microspore culture. Vestnik KazNU. Seriya Ekologicheskaya = Bulletin of the KNU: Environmental Series. 2013;38(2/2):
369-374.

Van Geyt J., Speckmann G.J., D’Halluin K., Jacobs M. In vitro induction
of haploid plants from unpollinated ovules and ovaries of
the sugarbeet (Beta vulgaris L.). Theor. Appl. Genet. 1987;73(6):
920-925. DOI 10.1007/BF00289399

Watts A., Kumar V., Bhat S.R. Centromeric histone H3 protein: from
basic study to plant breeding applications. J. Plant Biochem. Biotechnol.
2016;25:339-348. DOI 10.1007/s13562-016-0368-4.

Weich E.W., Levall M.W. Doubled haploid production of sugar beet
(Beta vulgaris L.). In: Maluszynski M., Kasha K.J., Forster B.P.,
Szarejko I. (Eds.) Doubled Haploid Production in Crop Plants.
New York: Springer Science + Business Media, 2003;255-263.
DOI 10.1007/978-94-017-1293-4_38.

Zheng M.Y. Microspore culture in wheat (Triticum aestivum) – doubled
haploid production via induced embryogenesis. Plant
Cell Tissue Organ Cult. 2003;73:213-230. DOI 10.1023/A:10230
76213639.

